# Medical Treatment of Hepatocellular Carcinoma

**DOI:** 10.4084/MJHID.2009.021

**Published:** 2009-12-16

**Authors:** Alessandro Granito, Luigi Bolondi

**Affiliations:** Department of Digestive Diseases and Interna Medicine, Policlinico S. Orsola Malpighi, Bologna, Italy

## Abstract

Hepatocellular carcinoma (HCC) is the fifth most common neoplasm and the third leading cause of cancer-related deaths worldwide. Cirrhosis, most often due to viral hepatitis, is the predominant risk factors for HCC and geographical differences in both risk factors and incidence are largely due to epidemiological variations in hepatitis B and C infection. Hepatic function is a relevant parameter in selecting therapy in HCC. The current clinical classification of HCC split patients into 5 stages, with a specific treatment schedule for any stage. As patients with early stages can receive curative treatments, such as surgical resection, liver transplantation or local ablation, surveillance program in high-risk populations has become mandatory. Sorafenib, a multikinase inhibitor, has recently shown survival benefits in patients at advanced stage of disease. Hopefully, new molecular targeted therapies and their combination with sorafenib or interventional and surgical procedures, should expand the therapeutic armamentarium against HCC.

## Introduction:

Hepatocellular carcinoma (HCC) currently ranks fifth in global cancer incidence representing the third cause of cancer-related death^1^.

In 70–90% of cases HCC develops on the background of cirrhosis or chronic liver inflammation (hepatitis) and it is currently the leading cause of death among cirrhotic patients^2^.

Risk factors and etiologies vary among geographical regions: chronic hepatitis C viral infection (HCV) represents the predominant risk factors in western countries and Japan, while chronic hepatitis B viral infection (HBV) is the main predisposing factor in Asia and Africa. Hepatitis B carriers are 100 times more likely to develop HCC than the uninfected persons with an annual incidence of 0.5% in non-cirrhotic cases and of 2–6% in cirrhotic patients 3,4 In patients who have established HCV-related cirrhosis the incidence of HCC is between 2%–8% per year^5^.

New in the epidemiology of HCC, especially in the west, is the emerging role of the metabolic syndrome, related to obesity and insulin resistance, as an important risk factor along with the well-established role of high consumption of alcohol^6^.

Differently from other neoplasms, HCC behaviour is rather peculiar with prognosis determined not only by the tumoral disease but also by the severity of the underlying liver disease. Until few years ago, HCC was a malignancy typically diagnosed at an advanced stage with a very poor prognosis. Nowadays, a wider range of therapeutic options can be offered including surgical resection, orthotopic liver transplantation (OLT), percutaneous ablation procedures, intra-arterial treatments and, more recently, molecular targeted therapies 7.

In addition, since it has well established that treatment is more effective when HCC is diagnosed at an early stage, efforts to improve diagnostic process and surveillance policy have assumed a major role in the management of HCC^8^. In this article we report a summary of the most recent information on novel advancements in the treatment of this neoplasm.

## Surveillance and Diagnosis:

The aim of HCC surveillance is to reduce mortality from the disease. A randomized controlled trial showed that HCC surveillance with liver ultrasound and serum alfafetoprotein (AFP) every 6 months improved survival in Chinese patients infected with HBV irrespective of the presence of cirrhosis^9^. Results from two european cohort studies confirm the potential benefits of this policy, especially in countries with high prevalence of disease^10^,^11^.

Surveillance for HCC should be performed using ultrasonography. Ultrasound has been reported to have a sensitivity of between 65% and 80% with a specificity greater than 90% when used as a screening test; this performance, at present, is superior to that of any of the available serologic tests^10^.

With regard to AFP, a value of 20 ng/mL has shown a good balance between sensitivity and specificity. However, at this level the sensitivity is only 60%, and therefore AFP alone should not be used for sceening^12^.

Proteomic research represents now a promising way but at present valid tumor markers have still not reached the clinical setting.

Surveillance with ultrasound every 6 months for detection of early HCC is recommended in cirrhotic patients and other specific risk groups^5^ (Table 1).

According to published guidelines, a diagnosis of HCC can be made in patients with cirrhosis who have a nodule greater than 2 cm identified on a dynamic imaging technique (CT scan, contrast ultrasound or MRI with contrast) with a typical vascular pattern (i.e., hyper-enhancement in the arterial phase with wash-out in the portal/venous phase)^5^,^13^. In patients with nodule greater than 2 cm but an atypical vascular pattern on imaging, a biopsy is recommended. Nodules between 1 and 2 cm presenting characteristic arterial enhancement features with venous wash-out on 2 different imaging modalities should be considered as HCC. Whether the typical vascular pattern is detected on a single imaging technique, a biopsy should be performed.

Nodules found on ultrasound surveillance that are smaller than 1 cm should be followed with ultrasound every 3 to 4 months to detect growth suggestive of malignant evolution.

If the hepatic lesion remains stable after 2 years, the patient can return to the standard surveillance of every 6 to 12 months.

In clinical practice the characterization of small hepatic nodules by imaging techniques remains difficult in a not negligible percentage of cases. Results from a previous study of this group have show that non-invasive criteria for diagnosis of HCC are satisfied in only 60% of small nodules. Moreover, it should be also considered that truly hypovascular HCC do exist, particularly in nodules of 1 to 2 cm where they reach the figure of 17% 14.

## Staging:

An accurate staging of the tumor before considering treatment of HCC is mandatory to estimate prognosis and decide which therapy may offer the greatest survival potential.

**Figure 1.**
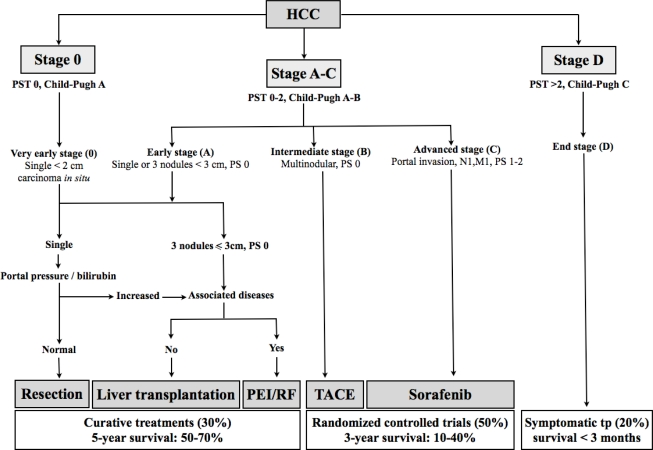
Barcelona Clinic Liver Cancer (BCLC) staging classification (modified from Bruix J and Sherman M^77^)

The so-called Barcelona-Clinic Liver Cancer (BCLC) is the most widely accepted staging system (Figure 1). It includes different variables (tumor stage, liver function, physical status, cancer related symptoms) and links staging with treatment modalities and with an estimation of life expectancy that is based on published response rates to the various treatments^15^.

This system stratifies patients into 5 stages that identify the ideal candidates for the therapies currently available. Patients with a single lesion less than 2 cm, who are asymptomatic and do not show vascular invasion or satellites represent the so-called “very early” HCC. This is a very well-differentiated HCC which correlates, from the pathological perspective, with the carcinoma “*in situ”* stage. The absence of microvascular invasion and dissemination offers the highest likelihood of cure. Results from Japanese studies have shown in these patients excellent outcome in terms of survival. Ninety percent and 71% after 5 years can be achieved with surgical resection or radiofrequency ablation (RFA), respectively with a recurrence rate of 8% at 3 years^16^,^17^. “Early-stage” disease is characterized by preserved liver function (Child-Pugh class A or B), and refers to HCC whitin the Milan criteria: single HCC of 5 cm or less, or up to 3 lesions less than 3 cm each, without vascular infiltration neither lymph node or distant metastases^18^. These patients can be effectively treated by therapies having curative intents such as surgical resection, liver transplantation (OLT) or local ablation, with a reported 5-year survival rates of 50–75%^18^,^19^,^20^.

The choice of therapy is not univocal and it should be based on severity of the liver dysfunction, presence of portal hypertension, medical comorbidities.

Patients with compensated cirrhosis and without HCC-related symptoms or vascular invasion that are outside of the criteria of “very early” or “early” stage correspond to the “intermediate” stage HCC. Untreated patients at this stage present a median survival of 16 months. Transarterial chemoembolization (TACE) raises the median survival of these patients to 19–20 months and is now regarded the standard care at this stage^21^.

Patients who have symptomatic tumors and/or invasive tumoral pattern (vascular invasion/extrahepatic spread) identify the “advanced” stage.

Until two years ago, lack of treatment options for patients at advanced stage produced a median survival of 6 months. Sorafenib, a multityrosine kinase inhibitor, has recently become the standard of care for these patients as it is the only treatment proven to prolong the survival in this setting^22^.

Patients with extensive tumor involvement leading to severe cancer-related symptoms and/or major impairment of liver function (Child–Pugh C) are considered as “end stage”. These patients have a life expectancy less than 3–6 months. They are not likely to benefit with any of the treatments aforementioned and should be treated only with symptomatic therapy.

## Treatment:

Treatment of HCC is multidisciplinary and involves hepatologists, oncologists, surgeons, and interventional radiologists. Several factors such as severity of underlying liver disease, tumor burden, physical status, associated diseases as well as availability and expertise in surgical and ablative therapies, should be considered before deciding therapeutic strategy.

Treatments for HCC have been conventionally divided into curative and palliative.

Curative treatments such as surgical resection, liver transplantation and percutaneous ablation, achieve complete responses in a high proportion of patients and are expected to improve survival. Palliative treatments are not aimed to cure but can obtain good response rates and also prolong survival in some cases.

Despite a wide diffusion of surveillance strategy among cirrhotic patients, only 30–40% of patients in western countries can undergo curative treatments^23^.

Resection is the treatment of choice for HCC in non-cirrhotic patients who represent however just 5% of cases in the west^24^.

In the absence of cirrhosis, surgical resection of this tumour is less likely to produce liver failure. Patients with HCC and concomitant cirrhosis are not all suitable for resection because of the risk for hepatic decompensation after surgical resection. Strict selection criteria are therefore required to avoid hepatic decompensation.

Nowadays this procedure is recommended only in patients with a single HCC and a well preserved liver function (Child Pugh A), with a normal bilirubin level and in the absence of portal hypertension (defined as the presence of oesophageal varices, platelet count of less than 100.000/μl or splenomegaly). Due to progress in surgical treatment, in these subjects survival rates at 5 years exceedes 70 % while treatment related mortality is less than 3% 25.

Tumor recurrence complicates 50% of cases at 3 years and 70% at 5 years after resection and are due to either growth of intrahepatic metastases understaged at pre-operative imaging studies or to the development of “*de novo”* tumors^20^,^26^,^27^,^28^.

Recurrence due to understaged HCC are usually observed within the first two years of follow-up, while metachronic tumours develop later.

Non-anatomical resection, presence of microscopic vascular invasion and histological differentiation, are well-known predictor of recurrence and survival^29^.

Thus, differently from percutaneous ablation, surgical resection can provides relevant informations about risk of recurrence and allows a risk-based enlistment for OLT.

Gene signature identification in liver tissue adjacent to the tumor, has been recently reported as promising tool to identify the patients at highest risk for recurrence of HCC, with the aim to adopt intensive clinical follow-up or chemopreventive strategies in such patients^30^.

The substantial recurrence rate has led to explore the potential benefit of adjuvant therapy, including systemic or hepatic-artery chemotherapy or chemoembolization, after resection. At present, however, definitive data and firm conclusions in this setting are still lacking^31^.

It has been shown that partial hepatectomy, when patients met the Milan criteria, allows a 5-year survival rate of 78.2%, as in the case of patients underwent OLT, a procedure which is instead dependent on availability of organs. Therefore, partial hepatectomy should be regarded as the appropriate strategy to treat well selected patients^32^.

Liver transplantation, in theory, would be the optimal therapeutic option for HCC as it simultaneously removes the tumor and underlying cirrhosis thus minimizing the risk of HCC recurrence. The best candidates for liver transplantation are those satisfying the Milan criteria. In these patients the survival rate after 5 years exceeded 70%, with a recurrence of less than 15%^18^,^19^. Organ allocation policy is based on the Model for End-Stage Liver Disease (MELD) score with the aim to transplant patients with the highest short-term risk of mortality. At present, the great limit of this therapeutic option is the organ shortage which increases waiting time and leads about 20% of patients to drop out before receiving the transplantation^19^,^33^. No definitive therapeutic strategies have been validated at present to delay tumor progression while listing. TACE, however, seem to reduce tumor burden and to delay progression even if it can precipitate liver failure^34^. Percutaneous ablation is cost-effective when waiting time is longer than 6 months but the risk of tumor seeding should not be disregarded^35^

The major questions currently related to liver transplantation for HCC are whether Milan criteria can be safely expanded, how to increase the scarce supply of donor organs, to assess the benefit of adjuvant therapies, such as percutaneous ablation and chemoembolization or systemic therapy, whilst patients are on the waiting list^36^–^45^.

Non-surgical treatment of hepatocellular carcinoma

Patients who are not candidates for surgical treatments, due to either tumor characteristics or underlying liver disease, can be considered for locoregional therapies, such as ablation, chemoembolization and radioembolization, or new therapies.

## Percutaneous ablation:

Because of several contraindications and the mortality and morbidity of surgery, alternative treatments have been developed. Percutaneous ablation include several minimally invasive techiniques consisting in the injection of chemicals (absolute ethanol, acetic acid, boiling saline) in the HCC nodule or its thermal destruction (radiofrequency, microwave, laser and cryoablation)^46^.

Generally it has performed under ultrasound guidance but it can be also done under CT or MRI control or during laparotomy or laparoscopy. These techiniques are inexpensive, require short hospitalization time and present rare complications^47^. Major contraindications are presence of ascites, severe hemostasis disorders (platelet count less than 50.000/mm^3^, prothrombin activity less than 50%), severe liver impairment, neoplastic vein thrombosis. 48 Treatment efficacy, defined as absence of contrast uptake on a dynamic imaging technique (CT scan, contrast ultrasound or MRI with contrast), can be evaluated one month after the procedure.

Percuteneous ablation, usually by radiofrequency ablation (RFA) and percutaneous ethanol injection (PEI), is the best therapeutic option in patients with early, unresectable HCC.^49^,^50^

PEI, the first percutaneous technique introduced in clinical practice, is widely available and a well-tolerated procedure with few side effects, but requires repeated injections on separated days. The rate of complete necrosis is closely correlated with tumor size; PEI has been reported to induce chemical coagulative necrosis in 70% to 80% of solitary HCC sized 3 cm or less and in almost 100% in tumors less than 2 cm; however, its efficacy drops to 50% in lesions between 3 and 5 cm likely because of the presence of septa inside the lesion that limit the spread of ethanol 46,48.

RFA achieves thermal coagulation using an alternating electric current. It produce a similar level of response as PEI in tumors less than 2 cm but with fewer sessions and may determine better results in tumors greater than 2 cm.^49^. According to guidilines of American Association for the Study of Liver Diseases (AASLD), RFA is the first option to be considered in tumors greater than 2 cm^51^,^52^. Moreover, a recent meta-analysis of randomized controlled trials comparing PEI with RFA has demonstrated that RFA offers a better 3-year survival than PEI (63% to 81% in RFA-treated patients versus 48% to 67% in PEI-treated patients)^52^.

The main limit of RFA is the rate of adverse events which is higher than that with ethanol. Mortality rates ranging from 0.1 to 0.3% and severe complication rate up to 10% 53–55. Major complications included peritoneal bleeding, pneumothorax, intestinal perforation, liver abscess, pleural effusion and bile duct stenosis^56^.

Specific contraindication for the use of RFA include: poorly differentiated tumors or subcapsular location due to a higher seeding risk, location of the tumor adjacent to the gastrointestinal tract, large bile ducts and large blood vessels^53^.

Recent studies have tried to compare PEI or RFA with surgical resection in paients with early HCC candidates to surgery. Although no definitive answer can be drawn, percutaneous ablation seem to achieve similar survival and disease-free survival than surgical resection, but with lower morbidity^57^–^60^. Similar to surgical resection, percutaneous ablation shows high rates of recurrence during follow-up that may reach 70% at 5 years^48^.

## Transarterial Chemoembolization:

Transarterial chemoembolization (TACE) is considered as the first line non-curative therapy for non-surgical patients with large/multifocal HCC who do not have vascular invasion or extrahepatic spread (BCLC intermediate stage). The rationale of this procedure is based on the knowledge of tumor vascularization: tumors > 2 cm mainly receive their blood supply from the end branches of hepatic artery and not from portal vein^61^. The complete procedure associates injection in the hepatic artery of a cytotoxic drug mixed with lipiodol (ethiodized oil), an oily contrast agent, followed by embolization usually by means of absorbable gelatin (Gelfoam) particles.

Cytotoxic drugs are preferentially delivered in the tumor and, when mixed with lipiodol, are progressively delivered inside the tumor. The most frequently used cytotoxic drugs are cisplatin and doxorubicin, which are amphiphilic and likely more retained in lipiodol, allowing an increased contact with tumoral tissue. Lipiodol remains embolized in the tumoral vessels and contributes, in addition to the embolizing agent, to ischemic necrosis of the tumor^48^.

The procedure should be as selective as possible to target just the tumor and to avoid ishemic damages to the surrounding liver parenchima.

Contraindications for TACE include absence of effective portal flow (portal vein thrombosis, hepatofugal flow in the portal vein, transjugular intrahepatic portosystemic shunt), liver decompensation (Child-pugh C), systemic infection, significant comorbidity (cardiac and renal failure), clotting disorders (platelets count < 50.000/mm^3^ or prothrombin activity <50%)^34^.

Controlled randomized studies and a cumulative meta-analysis have demonstrated that in patients with unresectable HCC, TACE achieve objective responses lasting 1 to 6 months in 35% (range 16% to 61%) of cases and determine a significant benefit, when compared with best supportive care, in terms of both reduced progression rate and improved survival^62^–^64^. These results have been fundamental to recommend TACE as the standard of care for patients in the Intermediate stage of the BCLC classification (fig.1). It must be oultlined, however, that about 90% of patients included in the RCT taken into account in the Llovet’s metanalysis are in the Child Pugh class A and that these trials were mostly performed in patienrs classified as “unresectabe”, including also early and advanced cases. Therefore the conclusions about widestread applicability of TACE in intermediate HCC, irrespective of Child Pugh A or B class should be critically revised.

TACE is associated with adverse events in approximately 10% of treated patients; these events include ischemic cholecystitis, nausea, vomiting, bone marrow depression, and abdominal pain. Moreover, a postembolization syndrome is reported in about 50% of patients treated with TACE and includes fever, abdominal pain, and moderate degree of intestinal obstruction. Treatment-related mortality is less than 5% 65.

Even if TACE is now the standard treatment for unresectable HCC, there is still not a standardized protocol for this procedure. Randomized trials are actually ongoing to compare different types of cytotoxic drugs, drug vehicles, and therapeutic schemes.

The potential benefit of TACE as neoadjuvant or adjuvant treatment in curative procedure, as well as the combined use of TACE with other procedures, are further issues under investigation.

Palliative treatment also include radiation therapy, both internal and external. The use of external radiation is however limited by the low radiation tolerance of the non-tumoral liver.

Better results, in terms of safety and disease control rates, are obtained with radioembolization, a brachytherapy that consists in delivering radioactive implants.

Microspheres containing radionuclide Yttrium-90 (Y-90), a pure β-emitter, are lodged via a catheter insertion into the hepatic artery that feed the tumors and emit local radiation with limited exposure to adjacent healthy tissue^66^.

Absolute contraindications for Y-90 radioembolization are: 1) hepatopulmonary shunt that would result in an harmful dose of radiation (> 30 Gy with a single infusion or > 50 Gy for multiple infusions) to the lungs, 2) the inability to prevent embolization of microspheres into the gastrointestinal tract, and 3) a history of previous external irradiation to the liver. Differently from TACE, due to an absent or minimal embolic effect, Y-90 radioembolization can be safely performed in patients with portal vein occlusion^67^.

Retrospective analyses and small non-controlled prospective studies have shown that Y-90 radioembolization results in high disease control rate with a median survival for advanced HCC cases of 12 months^68^–^70^. At present, however, no randomized trials comparing Y-90 radioembolization with locoregional, systemic therapies or best supportive care, have been published.

## New Therapies:

The management of patients with avanced HCC has been characterized for decades by limited therapeutic options since both hormonal compounds and conventional cytotoxic chemotherapy has failed to show a substantial benefit for patients with HCC^71^–^73^.

A better knowledge of molecular hepatocarcinogenesis and the following introduction of targeted agents that specifically act on the neoplastic pathways, have created a new therapeutic hope^74^.

Very recently, positive results have been reported with the use of sorafenib, an oral multikinase inhibitor, which inhibits tumor-cell proliferation and tumor angiogenesis and increases the rate of apoptosis in a wide range of tumor models^75^. A multicentric international phase III randomized controlled trial (the SHARP trial) has recently shown a 3-month survival improvement with a manageable toxicity in patients with advanced HCC in Chil-Pugh A cirrhotic stage. The median overall survival was 10.7 months with sorafenib and 7.9 months with placebo (hazard ratio for the sorafenib group 0.69, 95% CI 0.55–0.87, p=0.005). Median time to progression was 5.5 months with sorafenib versus 2.8 months with placebo (p<0.001)^22^.

The most common grade 3 drug-related adverse events observed in the SHARP study included diarrhoea and hand-foot skin reaction, both of which occurred in 8% of all patients treated with sorafenib

A survival benefit of sorafenib has been also shown in a subsequent randomized phase III study conducted in Asia, in patients with advanced HCC. In this study, the median overall survival increased from 4.1 months in the placebo group to 6.2 months in the sorafenib group (hazard ratio in the sorafenib group 0.67; 95% confidence interval, 0.49–0.93; P<0.0155)^76^.

As a consequence of these results, the indication to Sorafenib treatment is well established in Child A patients having advanced HCC with or without extrahepatic spread and vascular involvement.

Thus, any further drug being developed in this subgroup of HCC patients will have to be compared against the reference standard sorafenib.

## Future Perspectives:

Other settings where sorafenib is actually under evaluation are:

1. intermediate HCC (according to BCLC classification) in combination to TACE: most of patients at this stage were included in the SHARP trial and contributed to the significant result of this study;
2. Child B patients with advanced HCC: most patients in Child B have been treated in phase II and other studies, as well as in current clinical practice and the efficacy treatment and the frequency of adverse events seems to be similar to that of Child A patients, except perhaps for bilirubin increase;
3. Adjuvant setting after surgical or loco-regional treatment, and in combiunation with conventional treatments: these perspectives are currently under investigation in clinical trials, as well as combinations with other molecuartargeted agents and second line treatments in patients progresing under Sorafenib.

Drugs that specifically target key molecules in carcinogenesis have emerged over the last decade. The molecular targeting has become a promising approach for the effective treatment of various cancers, now including also hepatocellular carcinoma. Therefore, several studies evaluating the efficacy of other molecular therapies in HCC are currently at different stages of validation in phase I, II and III clinical trials. These new therapies include molecules or monoclonal antibody blocking different molecular targets which have been shown to play a crucial role in HCC proliferation such as vascular endothelial growth factor (VEGF), plateled-derived growth factor (PDGF), epidermal growth factor (EGF), EGF receptor (EGFR), and PI3K/Akt/mTOR signaling pathway^74^.

The demonstration of an altered expression of the target molecules should be mandatory in defining the inclusion criteria of future studies. This could help to define the beneficial effect in restricted but identifiable subgroups of patients and to correctly allocate patients to the specific treatment.

It is desirable that the positive results obtained by sorafenib represent a first step toward new, tumor biology-based, therapeutic chances. In this regards, it is as decisive identify predictive biomarkers to select patients more likely to benefit from any specific agent.

Most probably, major benefits will be obtained by combination of agents acting simultaneously on distinct molecular targets and bear great hopes for the treament of HCC in the next years.

## Figures and Tables

**Table 1.** Patients at risk for developing HCC who should be entered into surveillance programme (modified from Bruix J and Sherman M^77^).

Cirrhosis regardless of the aetiology High risk groups of hepatitis B carriers: asian males ≥40 years, asian females ≥50 years, family history of HCC, africans > 20 years, chronic B hepatitis with high HBV DNA levels and those with high degree of hepatic inflammatory activity. Hepatitis C Genetic hemochromatosis Patients with alpha1-antitrypsin deficiency, non-alcoholic steatohepatitis, autoimmune hepatitis have an increased risk of HCC. However, because of paucity of data, no recommendations for or against surveillance can be made.
